# The Frog Test: A Tool for Measuring Humor Theories' Validity and Humor Preferences

**DOI:** 10.3389/fnhum.2016.00040

**Published:** 2016-02-09

**Authors:** Ori Amir

**Affiliations:** Psychology Department, University of Southern CaliforniaLos Angeles, CA, USA

**Keywords:** humor theory, humor preferences, content analysis, humor, joke structure, joke, repetition, funniness

For over a century multiple theoretical accounts proposed different sets of necessary and sufficient conditions for distinguishing humorous from non-humorous stimuli. The theories ranged from Freud's (1960/1905) expression of forbidden thoughts, superiority theories (e.g., Gruner, 2000), and benign violations (McGraw and Warren, 2010) to incongruity resolution (Suls, 1972), error detection (Hurley et al., 2011), and even purely neural accounts (Biederman and Vessel, 2006; Amir et al., 2015). Proponents of the different theoretical accounts often show a high degree of conviction, suggesting introspection might not be the best tool for judging the validity of humor theories.

Other than introspection, two methods have been employed to test humor theories: content analysis and experimental approach. These methods have yielded much valuable insight, however they are imperfect. Content analyses examine a corpus of humorous stimuli in an attempt to determine whether the conditions for humor proposed by a theory are present in all corpus stimuli (e.g., Hurley et al., 2011). Unfortunately, the conditions proposed by many of the humor theories are too vague or abstract to allow a rigorous content analysis. Experimental approaches manipulate aspects of the joke and measure perceived funniness or its correlates (e.g., laughter, neural activity). Unfortunately, due to the complex, interdependent relationship between the elements of a joke, attempts to manipulate one element of a joke can result in a different joke on multiple levels.

I would like to propose a third approach which, while likely not resolving the dispute, might yield distinct insights: “the frog test.” An oft-cited metaphor, paraphrased from E. B. White (White and White, 1941, p. xvii)^1^ proclaims: “Analyzing humor is like dissecting a frog. Few people are interested and the frog dies of it.” I would like to suggest, stretching the metaphor, that if the surgeon is competent the frog might survive. That is to say, if a theory explains with some accuracy why a particular joke is funny, such explanation might not reduce the perceived funniness of the joke to the same extent an entirely invalid theoretical explanation might. That is because an invalid account would presumably distract its receivers' attention away from the elements of the joke they found humorous in the first place. Taking E. B. White's humorous metaphor as an example, the reason for its humorousness could be (among many other accounts):

1. The joy, Schadenfreude, or feelings of superiority over the death of the frog (e.g., superiority theory).
2. Highlighting the inappropriateness of attempting to explain a joke (error detection).

Let us assume, for the sake of argument, the second account is closer to the true reason for the joke's funniness. Suppose the joke is presented to two groups of participants. Each account is then offered to half of the participants as the reason why scientists believe the joke is funny. The joke is then repeated to the participants and they are asked to rate it for funniness. I predict the first, incorrect account for the joke's funniness would shift participants' attention away from the humorous element of the joke, so participants who were presented with that account would rate the joke as less funny subsequently, when it is repeated, relative to participants who received the more valid account.

The nature of the joke explanation effect can be further explored and controlled by asking participants to rate the explanation on various measures, e.g., How complex was the explanation? How well you understood it? How generic vs. joke-specific was it? How much do you agree with it? How much did you enjoy this explanation? How funny was the explanation itself? A baseline can be established with a control group who reads the joke twice, but in between, rather than a theoretical account of the joke they read unrelated text. The ratio of the funniness rating subsequent a particular theoretical account and the baseline score would compute the “frog test” score of that theoretical account.

While I propose the test as a potentially useful tool for judging the validity of humor theories, it might also provide a measure of individual differences in humor tastes. It might be that for some individuals with preference for an aggressive humor style the first account in the example above would be associated with higher funniness ratings, while for individuals with preference for intellectual humor the second account would enhance funniness (or rather reduce it to a lesser extent).

The “Frog Test” assumes reading a theoretical account of a joke would direct attention to—or enhance the internal representation of those elements or interpretations of the joke the theory deems pertinent. The assumption is in line with numerous studies demonstrating that context affects perceptual and linguistic processing so as to favor the context related aspects or meanings of a stimulus (e.g., context relevant meaning of an ambiguous word, Simpson, 1981; context relevant interpretation of an ambiguous image, Goolkasian and Woodberry, 2010). Moreover, it was demonstrated that while processing a joke, different meanings implied by the joke are activated at different times, suggesting the focus on different aspects of a joke or a particular interpretation of the joke can be subject to change (Vaid et al., 2003). That said, some theoretical accounts might predict a different outcome. For example Freudian theorists might argue that giving the correct account for a joke's funniness (e.g., a repressed desire to kill one's father) would forfeit the joke's effectiveness as a defense mechanism and might rather reduce funniness the most (while increasing anxiety). Such alternative explanations may be controlled for by the overall pattern of “Frog Scores” for different theories and by including in a regression, along with the Frog Score, additional measures of the reaction to the theoretical explanation (e.g., various ratings of it—as suggested above, galvanic skin response while processing it). So, for example, reading a Freudian theory's analysis of a joke might result in a relatively large reduction, on a subsequent reading of the joke, of its funniness ratings (i.e., a low Frog Score)—so far, that outcome is predicted by the Freudian theory and by the assumption of the Frog Test for the case that the Freudian theory is invalid. However, if other theoretical accounts of the joke that equally “miss the mark” would result in a similarly low Frog Score, and if other equally disturbing analyses of the joke result in similarly increased measures of anxiety, the overall pattern of results would go against the Freudian interpretation and suggest that the analysis is indeed invalid as implied by its low Frog Test score.

In summary, I propose a novel method for measuring the validity of theoretical accounts for humor and individual differences in humor preferences. The method relies on the assumption that presenting participants with theoretical accounts for a specific joke would shift their attention to those joke elements or the perspective the theory deems relevant. A unique advantage of the method is that it does not require a manipulation of joke content (as does the experimental approach) or determining whether vague or abstract theoretical conditions are met (as does content analysis). I do not propose the method is superior over the other approaches, rather it is a qualitatively different method, and as such, it might yield novel insights.

## Author contributions

The author confirms being the sole contributor of this work and approved it for publication.

### Conflict of interest statement

The author declares that the research was conducted in the absence of any commercial or financial relationships that could be construed as a potential conflict of interest.

## Footnotes

^1^E. B. White originally wrote: “Humor can be dissected, as a frog can, but the thing dies in the process and the innards are discouraging to any but the pure scientific mind.” The oft-used paraphrased version above arguably has a better joke structure, as the word that reveals the punch-line (“dies”) is near the sentence's end.
